# Supplementary material for: Simultaneous Expression of Different Therapeutic Genes by Infection with Multiple Oncolytic HSV-1 Vectors
Source: Biomedicines. 2024 Jul 16;12(7):1577. doi: 10.3390/biomedicines12071577 (PMC11274547; doi:10.3390/biomedicines12071577)
Supplement: Supplementary file 1 [file biomedicines-12-01577-s001.zip › biomedicines-3057367-supplementary.pdf]

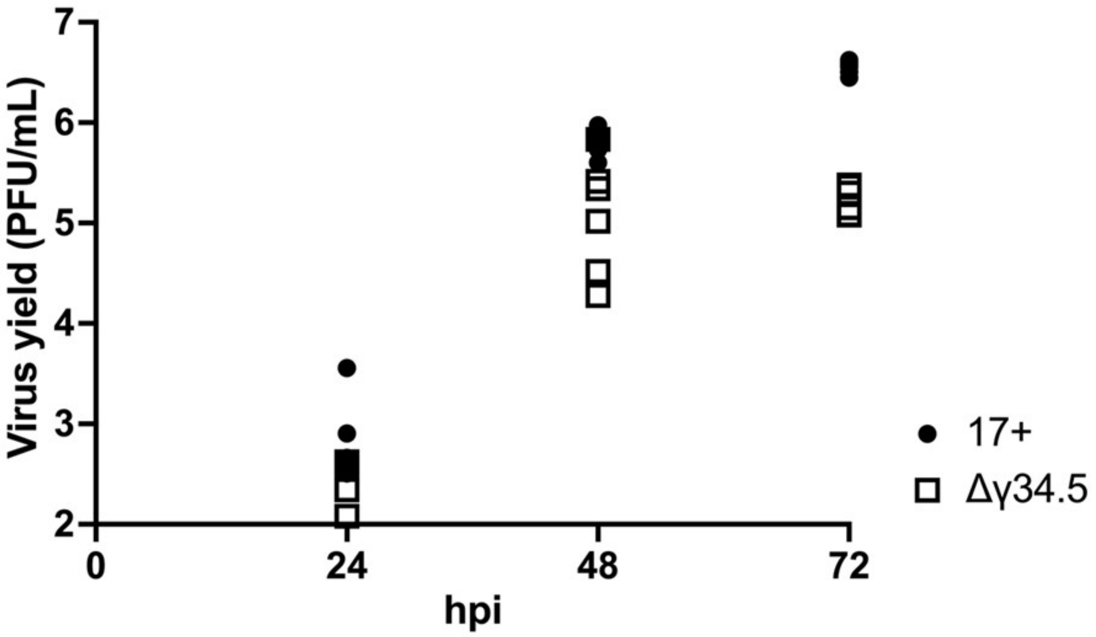

Figure S1: Δγ34.5 HSV-1 replicates in MDA-MB-231 cells less efficiently than wild-type HSV-1. MDA-MB-231 cells were infected with wild-type (17+) and γ34.5-deleted (Δγ34.5) HSV-1, as indicated (MOI = 0.01 PFU/cell). The graph displays the amounts of infectious particles released in the cell supernatants collected at different hpi, as shown. The experiment was performed three times and values ob-tained in each replicate are shown. Y axis is a logarithmic scale

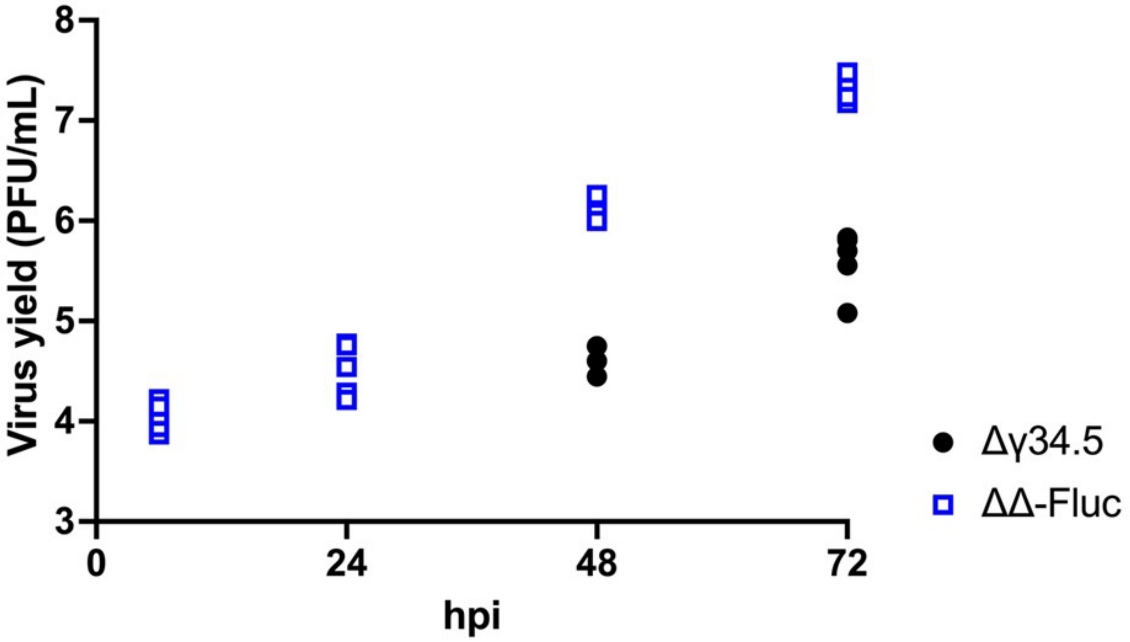

Figure S2:ΔΔ-Fluc replicates in MDA-MB-231 cells more efficiently than Δγ34.5. MDA-MB-231 cells were infected with Δγ34.5 and ΔΔ-Fluc, as indicated (MOI= 0.1 PFU/cell). The graph displays the amounts of infectious particles released in the cell supernatants collected at different hpi, as shown.

The experiment was performed three times and values obtained in each replicate are shown. Y axis is a logarithmic scale

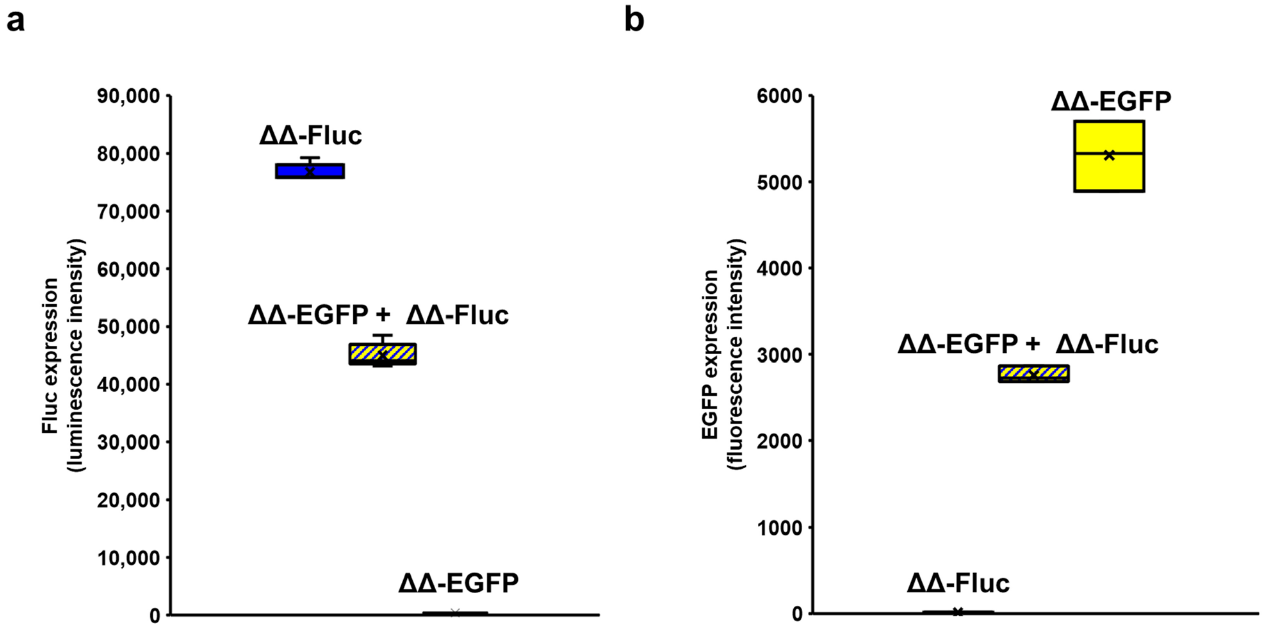

Figure S3: Viral particles harvested from mono- and mixed-infected MDA-MB-231 cells can infect Vero cells, leading to transgene expression. Supernatants harvested from MDA-MB-231 cells at 72 hpi with ΔΔ-Fluc, ΔΔ-EGFP, or ΔΔ-Fluc + ΔΔ-EGFP (each virus at an MOI of 1 PFU/cell in mono- and combined infections) were used to infect Vero cells. At 24 hpi, cells were lysed, and the intensity of Fluc (a) and EGFP (b) signals were evaluated. Displayed graphs show data from at least three independent experiments.
